# Overexpression of a panel of cancer stem cell markers enhances the predictive capability of the progression and recurrence in the early stage cholangiocarcinoma

**DOI:** 10.1186/s12967-020-02243-w

**Published:** 2020-02-10

**Authors:** Sureerat Padthaisong, Malinee Thanee, Nisana Namwat, Jutarop Phetcharaburanin, Poramate Klanrit, Narong Khuntikeo, Attapol Titapun, Sakkarn Sungkhamanon, Hideyuki Saya, Watcharin Loilome

**Affiliations:** 1 Department of Biochemistry, Faculty of Medicine, Khon Kaen University, 123 Mittraparp Road, Muang District, Khon Kaen, 40002 Thailand; 2 Cholangiocarcinoma Screening and Care Program (CASCAP), Khon Kaen University, Khon Kaen, 40002 Thailand; 3 Cholangiocarcinoma Research Institute, Faculty of Medicine, Khon Kaen University, Khon Kaen, 40002 Thailand; 4 Department of Surgery, Faculty of Medicine, Khon Kaen University, Khon Kaen, 40002 Thailand; 5 Department of Pathology, Faculty of Medicine, Khon Kaen University, Khon Kaen, 40002 Thailand; 6 Division of Gene Regulation, Institute for Advanced Medical Research (IAMR), Keio University School of Medicine, Tokyo, 160-8582 Japan

**Keywords:** Cholangiocarcinoma, Cancer recurrence, Cancer stem cell marker, Tumor marker, Prognostic factor

## Abstract

**Background:**

Cancer recurrence is the important problem of cholangiocarcinoma (CCA) patients, lead to a very high mortality rate. Therefore, the identification of candidate markers to predict CCA recurrence is needed in order to effectively manage the disease. This study aims to examine the predictive value of cancer stem cell (CSC) markers on the progression and recurrence of CCA patients.

**Methods:**

The expression of 6 putative CSC markers, cluster of differentiation 44 (CD44), CD44 variant 6 (CD44v6), CD44 variants 8-10 (CD44v8-10), cluster of differentiation 133 (CD133), epithelial cell adhesion molecule (EpCAM), and aldehyde dehydrogenase 1A1 (ALDH1A1), was investigated in 178 CCA tissue samples using immunohistochemistry (IHC) and analyzed with respect to clinicopathological data and patient outcome including recurrence-free survival (RFS) and overall survival (OS). The candidate CSC markers were also investigated in serum from CCA patients, and explored for their predictive ability on CCA recurrence.

**Results:**

Elevated protein level of CD44 and positive expression of CD44v6 and CD44v8-10 were significantly associated with short RFS and OS, while high levels of ALDH1A1 were correlated with a favorable prognosis patient. The elevated CD44v6 level was also correlated with higher tumor staging, whereas a decreasing level of ALDH1A1 was correlated with lower tumor staging. The levels of CD44, CD44v6 and CD44v8-10 were also correlated and were associated with a poor outcome. Furthermore, soluble CD44, CD44v6, CD44v8-10 and EpCAM were significantly increased in the recurrence group for early stage CCA; they also correlated with high levels of the tumor marker CA19-9. Elevated levels of CD44, CD44v6, CD44v8-10 or EpCAM alone or in combination has the potential to predict CCA recurrence.

**Conclusions:**

The overexpression of CD44, CD44v6, CD44v8-10 and EpCAM increases predictability of post-operative CCA recurrence. Moreover, the overexpression of the panel of CSC markers combined with CA19-9 could improve our predictive ability for tumor recurrence in early stage CCA patients. This result may be beneficial for the patients in order to predict the outcome after treatment and may be useful for clinical intervention in order to improve patient survival.

## Background

Cholangiocarcinoma (CCA) is the second most common primary hepatic cancer. It originates from the bile duct epithelium, accounting for 10–20% of primary liver cancers [1]. CCA can be divided into intrahepatic (iCCA), perihilar (pCCA) and distal (dCCA) forms based on their anatomical localization. iCCA arises from the bile duct epithelium inside the liver while pCCA and dCCA arise from epithelium outside of the liver [2]. Surgical resection is the only curative treatment and there is evidence suggesting that surgery with complete resection can improve patient survival [3]. In addition to surgery, adjuvant chemo- or radio-therapy is necessary to improve the patient’s outcome [4]. However, to date many patients experience recurrence after surgery resulting in the high mortality rate of CCA patients [5]. Therefore, understanding the tumor biology and the identification of markers to predict cancer recurrence are necessary to manage the disease.

Accumulated evidence suggests that subpopulations of cancer cells, called cancer stem cells (CSCs), show stem cell-like properties such as self-renewal. CSCs play a critical role in many cancer processes, including development, progression and recurrence [6]. Because CSCs impact tumor aggressiveness, CSC markers, which are the markers most commonly expressed in CSCs, become an important factor for predicting cancer progression and recurrence. Currently, several CSC markers have been established in CCA, including cluster of differentiation 44 (CD44), cluster of differentiation 133 (CD133), epithelial cell adhesion molecules (EpCAM), and aldehyde dehydrogenase 1 (ALDH1) [7]. The expression of these markers is usually associated with a poor clinical outcome in patients with different cancers [8–12]. CD44 is a cell surface glycoprotein with a single polypeptide chain. It functions as a cell surface receptor for hyaluronic acid. There are many CD44 variants (CD44v) generated by alternative splicing processes [13]. The expression of CD44 and its variant isoforms relates to tumor progression and recurrence in some cancers [11, 12, 14]. CD133, transmembrane glycoprotein, is another CSC marker for cancer stem-like cells in CCA. High expression of CD133 was reported to be significantly associated with more aggressive tumors and correlated with a worse outcome for cancer patients [15]. Another CSC marker, EpCAM, is mostly overexpressed in tumors of epithelial origin. EpCAM overexpression is usually associated with tumor progression, especially metastasis [16]. Moreover, it has long been recognized that EpCAM can be cleaved [17], and soluble EpCAM is also associated with the aggressive phenotype of tumors [18–20]. Even though surface markers are mostly used to isolate/characterize CSC, other types of markers have also been used to identify and predict tumor progression and patient outcome. ALDH1A1 is an enzyme belonging to the ALDH family that functions as a detoxifying enzyme and also converts retinol (vitamin A) into retinoic acid (RA) [10]. The overexpression of ALDH1A1 is mostly involved in poor cancer prognosis, however, numerous studies suggest that high expression of ALDH1A1 is also associated with a better prognosis of the patients [21]. Although many studies have reported that the expression of CD44, CD44v, CD133, EpCAM and ALDH1A1 is associated with tumor progression and can be used to predict patient’s outcome, their prognostic significance in the recurrence of CCA in patients has not been elucidated.

Therefore, in the present study, the expression of the above 6 putative CSC markers was investigated in 178 paraffin-embedded CCA tissue samples using immunohistochemical staining in order to explore their relationship with clinicopathological features and patient survival. Moreover, 4 candidate CSC markers were selected for further experimentation using enzyme-linked immunosorbent assay (ELISA) to examine their expression level in serum and to provide a potential CSC panel for predicting of CCA recurrence.

## Methods

### Patient selection and follow-up

Patients diagnosed with CCA and who underwent surgery at Srinagarind Hospital, Khon Kaen University, Khon Kaen, Thailand between February, 2007 and December, 2016 were retrospectively studied. Patients were excluded if they received either radiotherapy or chemotherapy before surgery in order to reduce the effect of neoadjuvant on protein expression. The patients were also excluded if they died within 30 days after surgery to avoid the effect of the operation. Tissue samples and pre-operative peripheral blood were obtained from patients and kept in the BioBank of the Cholangiocarcinoma Research Institute. All patients were assessed for clinicopathological characteristics including sex, age, tumor site, histology type, primary tumor (T stage), regional lymph node metastasis (N stage), distant metastasis (M stage), TNM stage, and post-operative chemotherapy status. In addition, pre-operative peripheral blood was used for laboratory testing including, tumor markers and liver function test.

For the follow-up protocol after surgery, the patients were examined every 3 months during the first year and every 6 months thereafter. Those patients who developed a new tumor after surgery were defined as having a post-operative recurrence. Overall survival (OS) was defined as the interval from the date of surgery to the time of death or until the last follow-up date, and recurrence-free survival (RFS) was defined as the interval from the date of surgery to the time of recurrence or until the last follow-up date.

### Immunohistochemistry (IHC) and grading

Two independent punctures from each patient were transfer onto a tissue microarray (TMA), and TMA was cut into 4 µm sections. IHC staining was performed to investigate protein expression. Tissue sections were de-paraffinized and rehydrated with stepwise xylene followed by 100%, 90%, 80% and 70% ethanol. Antigen retrieval was performed by microwave cooking with 10 mM sodium citrate buffer pH 6 and 0.05% Tween20 for 10 min. Tissue sections were treated with 0.3% hydrogen peroxide and 10% skim milk to block endogenous hydrogen peroxide activity and nonspecific binding for 30 min of each. Tissue sections was incubated with primary antibodies (CD44; #ab51037; dilution 1:50, CD44v6; #ab78960; dilution 1:50, CD133; #ab19898; dilution 1:100, EpCAM; #ab71916; dilution 1:100, ALDH1A1; #ab52492; dilution 1:100) (Abcam, UK), and (CD44v8-10; #LKG-M001; dilution 1:50) (Cosmo Bio, JP) for 1 h at room temperature followed by 4 °C overnight. Sections were washed in phosphate buffered saline (PBS) with 0.1% tween20 and incubated with secondary antibody (Dako EnVision) for 1 h, except CD44v8-10 to which was added anti-rat antibody (#ab6734; (Abcam, UK)) and left for 3 h. The signal was developed with a 3,3′diaminobenzidine tetrahydrochloride (DAB) substrate kit (Vector Laboratories, Inc., CA) for 5–10 min. Sections was counterstained with Mayer’s hematoxylin for 2 min and dehydrated stepwise with 70%, 80%, 90%, 100% ethanol and xylene. Next, sections were mounted with permount and observed under a light microscope by two independent observers in a blinded manner. Inconsistent data were discussed by the observers until a final agreement was reached.

Protein expression was analyzed according to staining frequency and intensity. The staining frequency of the protein was semi-quantitatively scored based on the percentage of positive cells, 0% = negative, 1–25% = + 1, 26–50% = + 2, and > 50% = + 3. The intensity of protein expression was scored as weak = 1, moderate = 2, and strong = 3. The final immunohistochemical score was determined by multiplying the intensity scores with the frequency scores, with a minimum score of 0 and a maximum of 9. The average score of each patient was calculated from two independent punctures. Finally, the median value was calculated by grading the scores of all patients. This was used as a cut-off point with the patients having a grading score lower than the median being classified as the low expression group, while those with a grading score equal to or higher than the median were classified as the high expression group [22]. For the proteins which have a median equal to zero, the patients have a grading score equal to zero, being classified as the negative group, while those with a grading score above zero are classified as the positive group.

### Enzyme-linked immunosorbent assay (ELISA)

Indirect ELISA was used to examine soluble protein levels in patient serum. Firstly, serum was diluted in coating buffer (dilution 1:100). Plates were coated with 100 µl of serum and incubated at 4 °C for 18 h. Then, the plates were blocked with 200 µl of 5% skim milk and incubated at 37 °C for 1 h. After washing, 100 µl of primary antibodies (CD44; #ab2212; dilution 1:200) (Abcam, UK), (CD44v6; AB2080; dilution 1:800) (Merck, Germany), (CD44v8-10; #LKG-M001; dilution 1:10,000), and (EpCAM; #MA1-46104; dilution 1:200) (Thermo Fisher Scientific, USA) were added and incubated at 37 °C for 2 h. Then, the excess antibody was washed off and 100 µl of HRP-conjugated secondary antibodies were added (anti-mouse antibody for CD44; #A16166; dilution 1:400; (Thermo Fisher Scientific, USA), anti-rabbit antibody for CD44v6; #G21234; dilution 1:1000; (Invitrogen, USA), anti-rat antibody for CD44v8-10, #ab6734; dilution 1:25,000 (Abcam, UK), anti-mouse antibody for EpCAM, #A16166, dilution 1:400; (Thermo Fisher Scientific, USA)). After incubation, the excess antibody was washed off and the signal was developed using 100 µl of 1 mg/ml OPD substrate for 30 min. The reaction was stopped with 100 µl of 4 N H_2_SO_4_ reagent and the OD measured on an ELISA reader at 492 nm.

### Statistical analyses

Statistical analyses were carried out using the Statistical Package for the Social Sciences; SPSS software v.17. The association between protein expression and the clinicopathological features of the CCA patients was determined using the Chi square test. Overall and recurrence-free survival analyses were performed using Kaplan–Meier (log-rank) analysis. The correlation between protein types was analyzed using Pearson’s correlation. The different of IHC score in different staging was analyzed using Kruskal–Wallis Test. The results from ELISA were analyzed by student’s *t* test. The receiver operator characteristic curve (ROC curve) and logistic regression were used to determine the predictive ability with respect to cancer recurrence of soluble protein levels or the combination with tumor markers. A *p*-value less than 0.05 was considered as statistically significant.

## Results

### Characteristics of CCA patients

A total of 178 CCA samples were studied with 64 cases (36%) from females and 114 (64%) from males. The age ranged between 42 and 82 years (median = 61 years). 96 cases (54%) were intrahepatic CCA and 82 (46%) were extrahepatic CCA. The histology type was characterized as papillary 83 cases (47%) and other 95 cases (53%). The staging was classified according to TNM staging. 109 cases (61%) were classified as primary tumor (T) stage I, II and 69 cases (39%) were T stage III, IV. Regional lymph nodes (N) and distant (M) metastases were also characterized. Among 178 patients, 72 cases (40%) had regional lymph node metastasis but only 4 cases (2%) showed distant metastasis. 80 cases (45%) were divided into early stage (TNM stage I, II) while 98 cases (55%) were late or advanced stage (TNM stage III, IV) (Table 1).

**Table 1:** Clinicopathological characteristics of CCA patients and the correlation with 6 putative CSC marker expression

Variables	n (178)	CD44		*p*	CD44v6		*p*	CD44v8-10		*p*	CD133		*p*	EpCAM		*p*	ALDH1A1		*p*
		Low	High		Neg.	Pos.		Neg.	Pos.		Neg.	Pos.		Low	High		Low	High	
Sex																			
Female	64	29	35		33	31		31	33		37	27		28	36		34	30	
Male	114	33	81	*0.028*	78	36	*0.026*	72	42	0.056	77	37	0.194	57	57	0.423	60	54	0.950
Age (year)																			
Less than 61	85	33	52		54	31		49	36		54	31		44	41		43	42	
61 or greater	93	29	64	0.285	57	36	0.758	54	39	0.955	60	33	0.891	41	52	0.306	51	42	0.570
Tumor site																			
Intrahepatic	96	33	63		49	47		52	44		54	42		40	56		48	48	
Extrahepatic	82	29	53	0.890	62	20	*0.001*	51	31	0.280	60	22	*0.019*	45	37	0.079	46	36	0.417
Histology type																			
Papillary	83	32	51		53	30		46	37		55	28		37	46		40	43	
Others	95	30	65	0.330	58	37	0.700	57	38	0.537	59	36	0.564	48	47	0.428	54	41	0.249
Primary tumor (T)																			
I, II	109	44	65		75	34		65	44		70	39		51	58		53	56	
III, IV	69	18	51	0.051	36	33	*0.026*	38	31	0.548	44	25	0.951	34	35	0.746	41	28	0.160
Lymph nodes (N) metastasis																			
No	106	39	67		76	30		66	40		68	38		52	54		45	61	
Yes	72	23	49	0.505	35	37	*0.002*	37	35	0.149	46	26	0.971	33	39	0.673	49	23	*0.001*
Distant metastasis (M)																			
No	174	59	115		107	67		102	72		112	62		83	91		90	84	
Yes	4	3	1	0.088	4	0	0.116	1	3	0.178	2	2	0.554	2	2	0.927	4	0	0.056
TNM stage																			
I, II	80	32	48		59	21		51	29		51	29		40	40		32	48	
III, IV	98	30	68	0.191	52	46	*0.005*	52	46	0.151	63	35	0.941	45	53	0.588	62	36	*0.002*

*CD44* cluster of differentiation 44, *CD44v* CD44 variant, *CD133* cluster of differentiation 133, *EpCAM* epithelial cell adhesion molecules, *ALDH1A1* aldehyde dehydrogenase 1A1, *TNM* primary tumor-node-metastasis, *Neg* negative expression, *Pos* positive expression

### Correlation between CSC marker expression and clinicopathological features

**Fig. 1:**
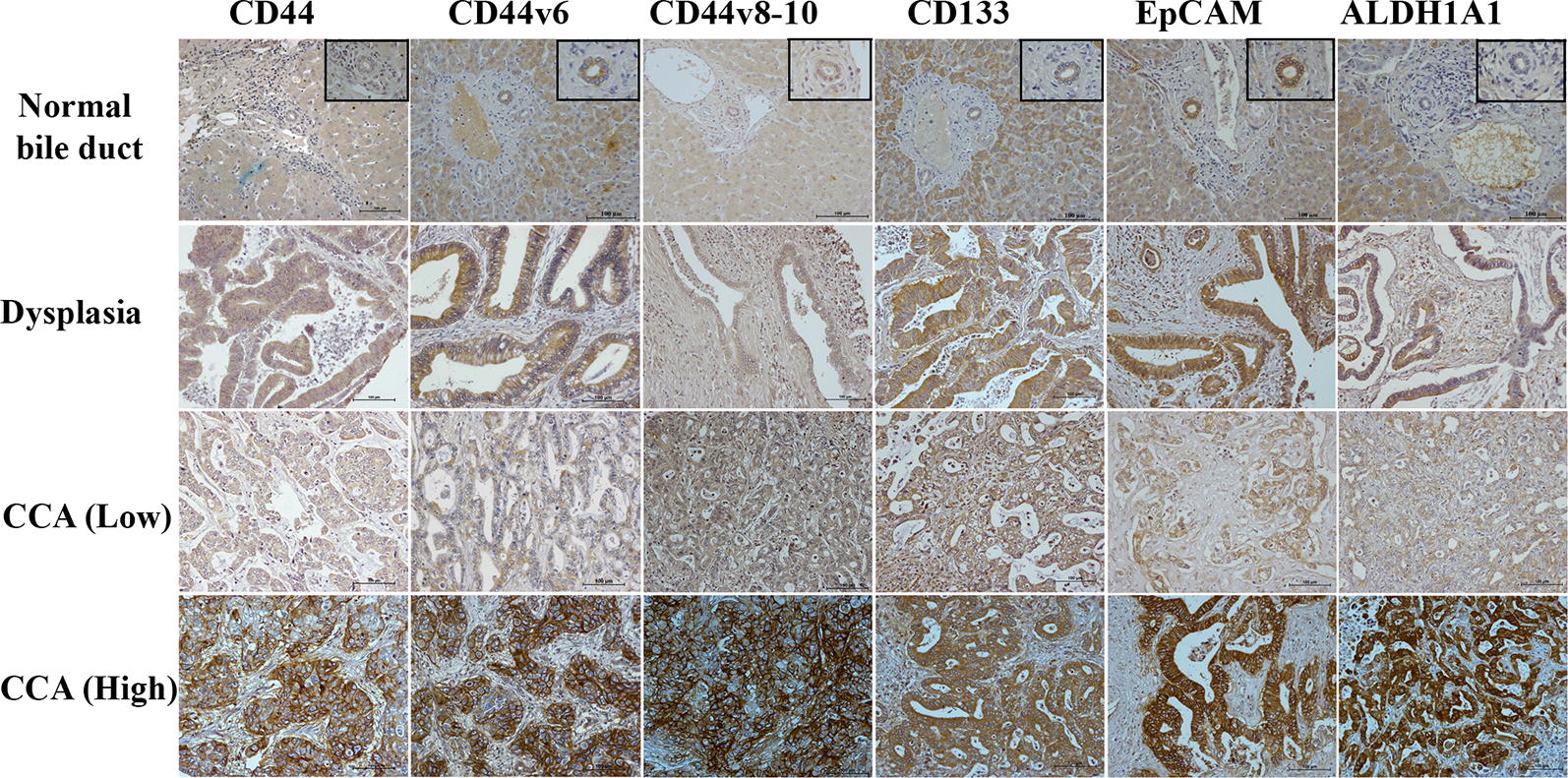
Representative immunohistochemical stanning of CD44, CD44v6, CD44v8-10, CD133, EpCAM, and ALDH1A1 in normal bile duct, dysplasia and CCA. For CCA, negative or low membrane expression of CD44, CD44v6, CD44v8-10, CD133, EpCAM are shown in the upper panel and positive and high membrane expression in the lower panel, low cytoplasmic expression of ALDH1A1 is shown in the upper panel and high cytoplasmic expression in the lower panel

The expression levels of the CSC marker were investigated using immunohistochemistry. The representative figures of a normal bile duct, the precancerous (dysplasia) stage and CCA are shown in Fig. 1. To investigate the correlation between protein expression and clinicopathological features, the expression of CSC markers was divided into those with low and those with high expression and also those with negative and those with positive expression. High expression of candidate CSC markers CD44, EpCAM, ALDH1A1 and positive expression of CD44v6, CD44v8-10, and CD133 were 65%, 52%, 47%, 38%, 42%, and 36%, respectively. High expression of CD44 and positive expression of CD44v6 was observed mostly in males (*p* = 0.028 and *p *= 0.026). In addition, positive CD44v6 and CD133 expression was frequently observed in intrahepatic CCA (*p* = 0.001 and *p *= 0.019). A significant association was found between T stage and CD44v6 expression (*p* = 0.026). Additionally, reginal lymph node metastasis and TNM staging were significantly associated with CD44v6 (*p* = 0.002 and *p* = 0.005) and ALDH1A1 expression (*p* = 0.001 and *p *= 0.002) (Table 1).

### The prognostic significance of clinicopathological features

To identify prognostic factors for CCA patients, we analyzed all clinicopathological features including sex, age, tumor site, histology type, T stage, regional lymph node metastasis and distant metastasis status, TNM stage, post-operative chemotherapy (CMT) status with recurrence-free survival (RFS) and overall survival (OS) of the patients. The median RFS and OS were 15 and 17 months, respectively. Among all clinicopathological features, we found that patients with a higher T stage, regional lymph nodes and higher TNM staging were significantly correlated with shorter RFS compared with patients with a low T stage, absent regional lymph nodes, or low TNM stage (*p* < 0.001, *p* = 0.001 and *p* < 0.001, respectively). The results for OS analysis also showed a similar result, except that patients with age equal to 61 or greater were also significantly associated with a short OS (*p* = 0.032). There was no significant correlation between sex, histology type, tumor site, distant metastasis status, and post-operative CMT status with RFS and OS (Table 2).

**Table 2:** Univariate analysis of factors predicting recurrence-free and overall survival

Variable	Median RFS (months)	Recurrence-free survival		Median OS (months)	Overall survival		
		HR	*P* (95% CI)		HR	*P* (95% CI)	
Overall	15.0			17.0			
Sex							
Female	13.0	1	0.336 (0.853–1.593)	16.9	1	0.270 (0.872–1.634)	
Male	15.0	1.166		17.4	1.193		
Age (year)							
Less than 61	16.0	1	0.208 (0.899–1.630)	19.2	1	*0.032* (1.028–1.876)	
61 or greater	13.0	1.211		16.3	1.388		
Tumor site							
Intrahepatic	12.5	1	0.449 (0.664–1.199)	16.0	1	0.560 (0.680–1.232)	
Extrahepatic	16.0	0.892		18.5	0.915		
Histology type							
Papillary	16.0	1	0.253 (0.884–1.599)	17.6	1	0.759 (0.779–1.409)	
Others	13.0	1.189		16.3	1.047		
Primary tumor (T)							
I, II	18.0	1	*< 0.001* (1.956–3.771)	22.0	1	*< 0.001* (1.907–3.629)	
III, IV	9.0	2.716		11.0	2.630		
Reginal lymph nodes (N) metastasis							
No	17.0	1	*0.001* (1.265–2.362)	20.0	1	*< 0.001* (1.442–2.704)	
Yes	10.5	1.729		13.0	1.975		
Distant metastasis (M)							
No	15.0	1	0.762 (0.432–3.150)	17.0	1	0.519 (0.513–3.751)	
Yes	12.0	1.166		13.5	1.387		
TNM stage							
I, II	18.0	1	*< 0.001* (1.576–2.986)	21.6	1	*< 0.001* (1.711–3.255)	
III, IV	10.0	2.169		12.8	2.360		
Post-operative CMT							
No	15.0	1	0.933 (0.727–1.340)	16.9	1	0.556 (0.671–1.239)	
Yes	15.0	0.987		19.0	0.912		
CD44							
Low	18.0	1	*0.007* (1.129–2.170)	22.0	1	*0.001* (1.250–2.399)	
High	13.0	1.565		16.1	1.732		
CD44v6							
Neg.	17.0	1	*0.001* (1.234–2.311)	19.2	1	*0.006* (1.128–2.081)	
Pos.	11.0	1.689		12.8	1.532		
CD44v8-10							
Neg.	*16.0 *	1	*0.007* (1.125–2.072)	*20.0*	1	*< 0.001 *(1.271–2.352)	
Pos.	13.0	1.527		14.1	1.729		
CD133							
Neg.	16.0	1	0.103 (0.949–1.765)	18.1	1	0.083 (0.965–1.794)	
Pos.	12.5	1.295		16.1	1.316		
EpCAM							
Low	14.0	1	0.135 (0.594–1.073)	16.3	1	0.261 (0.629–1.134)	
High	16.0	0.798		18.6	0.844		
ALDH1A1							
Low	11.0	1	0.075 (0.569–1.028)	16.0	1	*0.022* (0.525–0.950)	
High	17.0	0.765		19.9	0.706		

*TNM* primary tumor-node-metastasis, post-operative, *CMT* post-operative chemotherapeutic treatment, *HR* hazard ratio, *95% CI* 95% confidence interval, *CD44* cluster of differentiation 44, *CD44v* CD44 variant, *CD133* cluster of differentiation 133, *EpCAM* epithelial cell adhesion molecules, *ALDH1A1* aldehyde dehydrogenase 1A1, *TNM* primary tumor-node-metastasis, *Neg* negative expression, *Pos* positive expression

### The expression of CSC markers and their prognostic significance in CCA patients

The expression of candidate CSC markers CD44, CD44v6, CD44v8-10, CD133, EpCAM, and ALDH1A1 was analyzed with respect to RFS and OS. Univariate analysis showed that the patients with a high expression of CD44, positive expression of CD44v6 and CD44v8-10 had a shorter RFS compared with other patients (*p* = 0.007, *p* = 0.001 and *p* = 0.007, respectively). In addition, a high expression of CD44 and ALDH1A1, positive expression of CD44v6 and CD44v8-10 was associated with a shorter OS compared with the other group of patients (*p* = 0.001, *p* = 0.022, *p* = 0.006 and *p* < 0.001, respectively) (Table 2). Moreover, multivariate analysis showed that CD44 and CD44v8-10 could be used as prognostic factors independent of clinicopathological characteristics for RFS (*p* = 0.020 and *p* = 0.012) (Table 3) and OS (*p* = 0.002 and *p* = 0.001) (Table 4).

**Table 3:** Multivariate analysis of factors predicting recurrence-free survival

Variable	Recurrence-free survival		
	HR	95% CI	*p*
Model A			
Primary tumor (T) (III/IV vs I/II)	2.404	1.537–3.759	*< 0.001*
Lymph nodes metastasis (N) (Yes vs No)	1.451	0.944–2.229	0.090
TNM Stage (III/IV vs I/II)	1.025	0.589–1.783	0.931
CD44 (High vs Low)	1.476	1.062–2.051	*0.020*
Model B			
Primary tumor (T) (III/IV vs I/II)	2.341	1.482–3.697	*< 0.001*
Lymph nodes metastasis (N) (Yes vs No)	1.368	0.880–2.126	0.164
TNM Stage (III/IV vs I/II)	1.024	0.579–1.871	0.934
CD44v6 (Pos. vs Neg.)	1.350	0.975–1.871	0.071
Model C			
Primary tumor (T) (III/IV vs I/II)	2.665	1.686–4.212	*< 0.001*
Lymph nodes metastasis (N) (Yes vs No)	1.441	0.934–2.223	0.098
TNM Stage (III/IV vs I/II)	0.933	0.528–1.649	0.810
CD44v8-10 (Pos. vs Neg.)	1.491	1.092–2.036	*0.012*

*TNM* size of primary tumor-node metastasis-distant metastasis, *CD44* cluster of differentiation 44, *CD44v* CD44 variant, *Neg* negative expression, *Pos* positive expression

**Table 4:** Multivariate analysis of factors predicting overall survival

Variable	Overall survival		
	HR	95% CI	*p*
Model A			
Age less (61 or greater vs than 61)	1.429	1.051–1.944	*0.023*
Primary tumor (T) (III/IV vs I/II)	2.370	1.517–3.702	*< 0.001*
Lymph nodes metastasis (N) (Yes Vs No)	1.753	1.129–2.723	*0.012*
TNM stage (III/IV vs I/II)	1.013	0.576–1.782	0.964
CD44 (high vs low)	1.701	1.221–2.371	*0.002*
Model B			
Age less (61 or greater vs than 61)	1.510	1.113–2.050	*0.008*
Primary tumor (T) (III/IV vs I/II)	2.321	1.474–3.654	*< 0.001*
Lymph nodes metastasis (N) (Yes vs No)	1.706	1.086–2.682	0.021
TNM stage (III/IV vs I/II)	0.987	0.554–1.759	0.964
CD44v6 (Pos. vs Neg.)	1.168	0.848–1.609	0.340
Model C			
Age less (61 or greater vs than 61)	1.493	1.100–2.027	*0.010*
Primary tumor (T) (III/IV vs I/II)	2.765	1.739–4.395	*< 0.001*
Lymph nodes metastasis (N) (yes vs no)	1.818	1.167–2.833	*0.008*
TNM Stage (III/IV vs I/II)	0.827	0.461–1.485	0.525
CD44v8-10 (Pos. vs Neg.)	1.694	1.234–2.326	*0.001*
Model E			
Age less (61 or greater vs than 61)	1.509	1.112–2.047	*0.008*
Primary tumor (T) (III/IV vs I/II)	2.411	1.545–3.764	*< 0.001*
Lymph nodes metastasis (N) (yes vs no)	1.724	1.104–2.691	*0.017*
TNM stage (III/IV vs I/II)	0.955	0.539–1.693	0.875
ALDH1A1 (high vs low)	0.867	0.636–1.182	0.366

*TNM* size of primary tumor-node metastasis-distant metastasis, *CD44* cluster of differentiation 44, *CD44v* CD44 variant, *ALDH1A1* aldehyde dehydrogenase 1A1, *Neg* negative expression, *Pos* positive expression

**Fig. 2:**
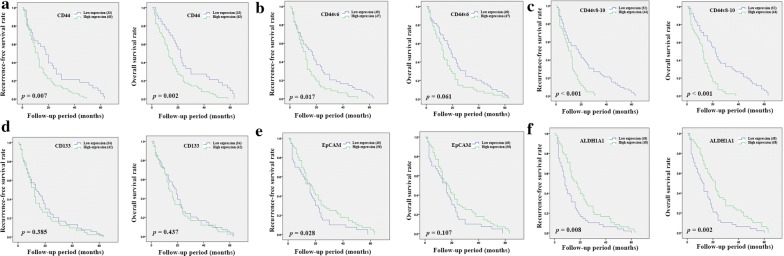
Kaplan–Meier analyses of recurrence-free and overall survival of CD44 (**a**), CD44v6 (**b**), CD44v8-10 (**c**), CD133 (**d**), EpCAM (**e**), and ALDH1A1 (**f**) in intrahepatic CCA patients

**Fig. 3:**
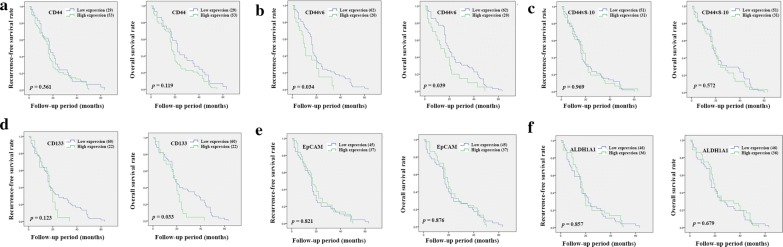
Kaplan–Meier analyses of recurrence-free and overall survival of CD44 (**a**), CD44v6 (**b**), CD44v8-10 (**c**), CD133 (**d**), EpCAM (**e**), and ALDH1A1 (**f**) in extrahepatic CCA patients

Kaplan–Meier analysis was used to examine the importance of tumor location. The result from intrahepatic CCA showed that a high expression of CD44 or the positive expression of CD44v6, and CD44v8-10 was significantly correlated with a shorter RFS compared with samples showing a low expression (*p* = 0.007, *p* = 0.017 and *p* < 0.001, respectively), while a high expression of EpCAM and ALDH1A1 was significantly correlated with a favorable prognosis in patients (*p* = 0.028 and *p* = 0.008) (Fig. 2). The results from OS analysis showed that patients with a high expression of CD44 or a positive expression of CD44v8-10 or a low expression of ALDH1A1 also had a shorter OS compared with other groups (*p* = 0.002, *p* < 0.001 and *p* = 0.002, respectively) (Fig. 2). The result from extrahepatic CCA showed that a positive expression of CD44v6 was significantly correlated with a shorter RFS and OS (*p* = 0.034 and *p* = 0.039) (Fig. 3). Additionally, a positive expression of CD133 was significantly correlated with a shorter OS compared with samples showing a low expression (*p* = 0.033) (Fig. 3).

**Fig. 4:**
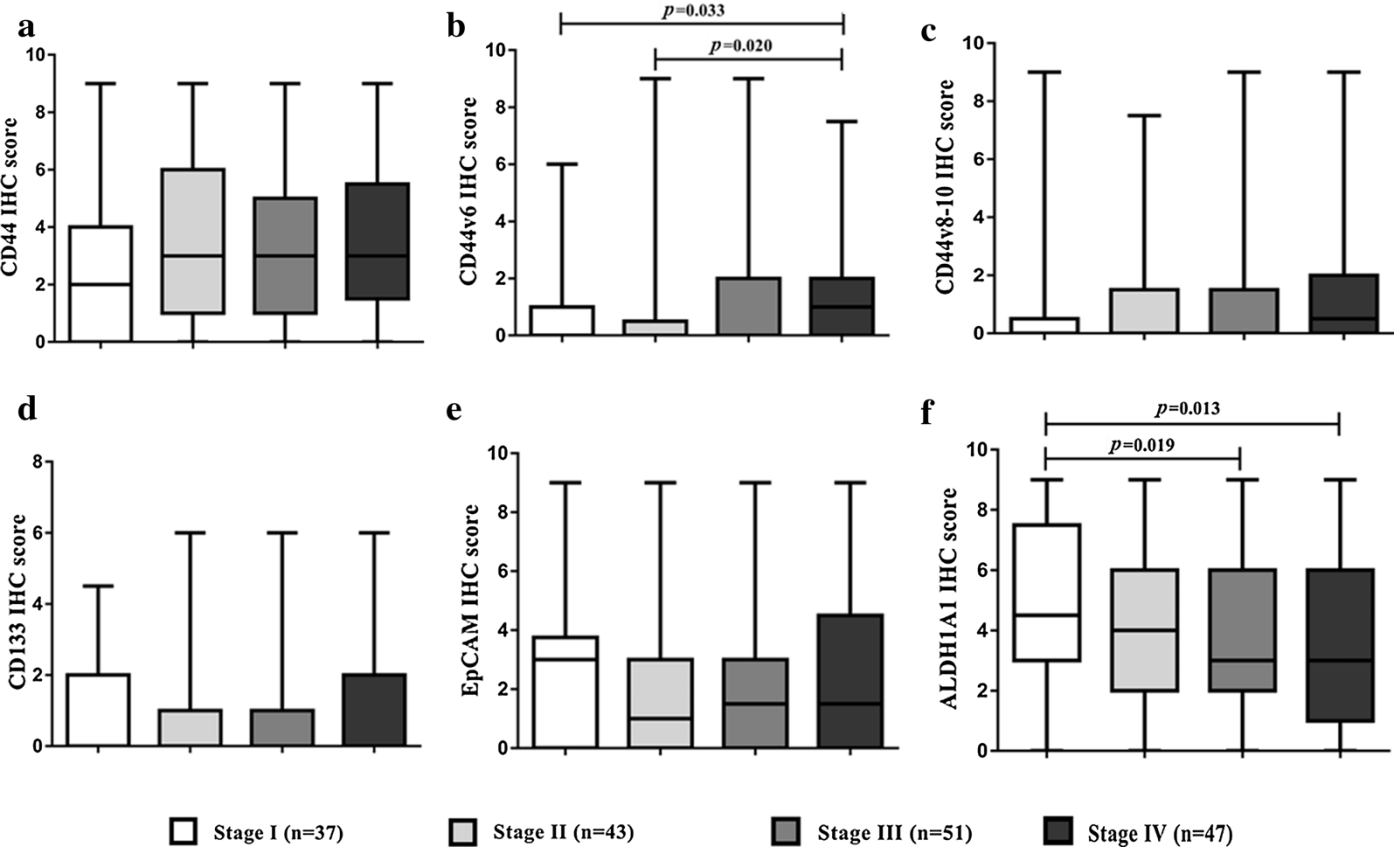
The different IHC scores for the various protein types, CD44 (**a**), CD44v6 (**b**), CD44v8-10 (**c**), CD133 (**d**), EpCAM (**e**), and ALDH1A1 (**f**) according to tumor staging

In addition to the DFS and OS analyses, the differences in IHC scores for different protein types were evaluated for different tumor stages. The expression of CD44, CD44v6, and CD44v8-10 seems to increase at higher stages compared with stage I tumor (Fig. 4a–c). Significant differences were observed between stages I and IV, II and IV of CD44v6 (*p* = 0.033 and *p* = 0.020, respectively) (Fig. 4b). In addition, the expression level of ALDH1A1 could be used to classify tumor staging. We found that ALDH1A1 expression level decreased along with tumor staging and was significantly decreased in stages III and IV compared with stage I tumor (*p* = 0.019 and *p *= 0.013) (Fig. 4f).

**Fig. 5:**
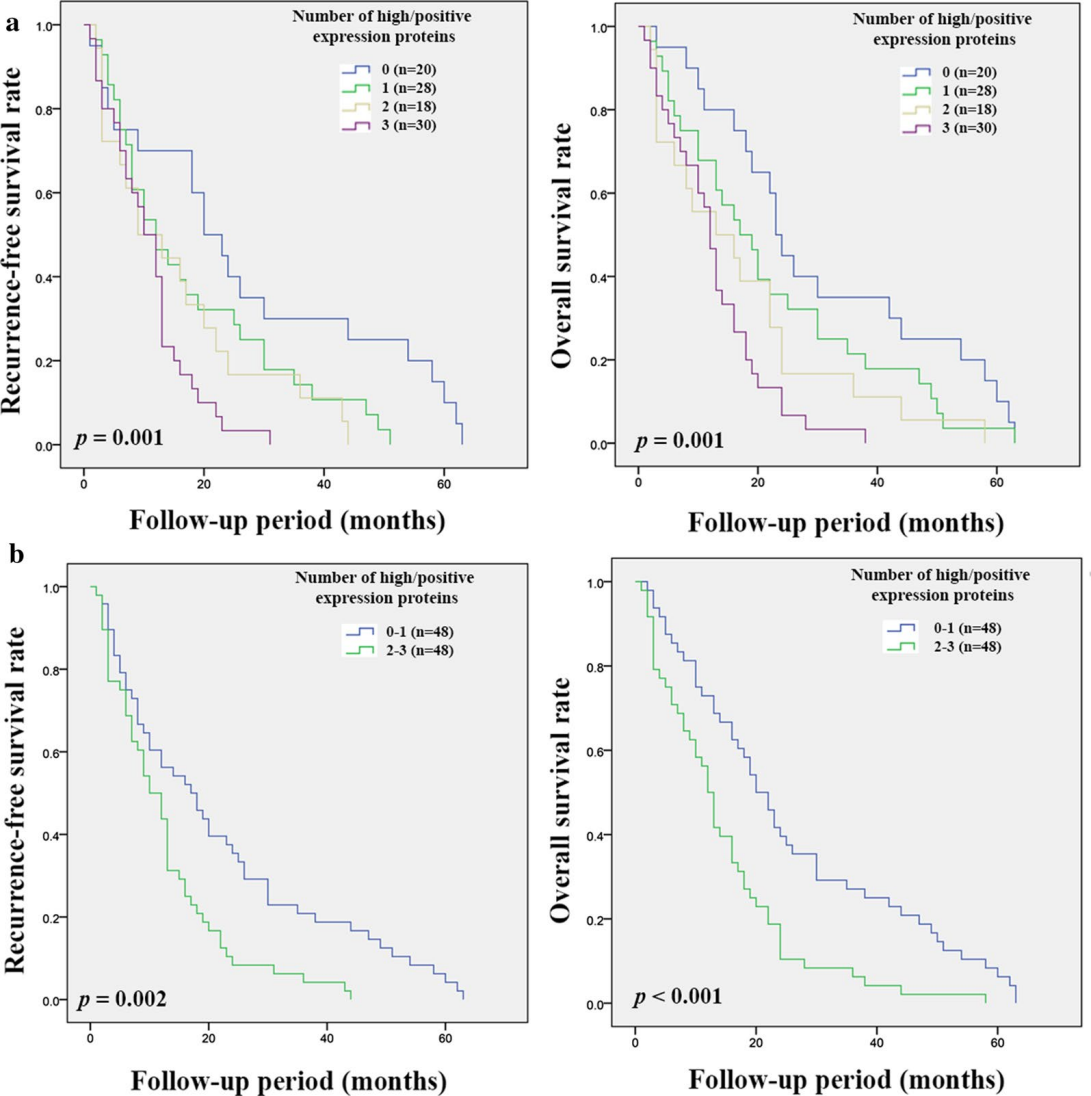
Kaplan–Meier analyses of recurrence-free and overall survival on the combination of CD44, CD44v6, and CD44v8-10 expression in intrahepatic CCA patients. **a** Recurrence-free and overall survival of patients according to the number of high or positive expression proteins. **b** Recurrence-free and overall survival of patients with zero or one high or positive expression marker versus high or positive expression of two or three markers

**Fig. 6:**
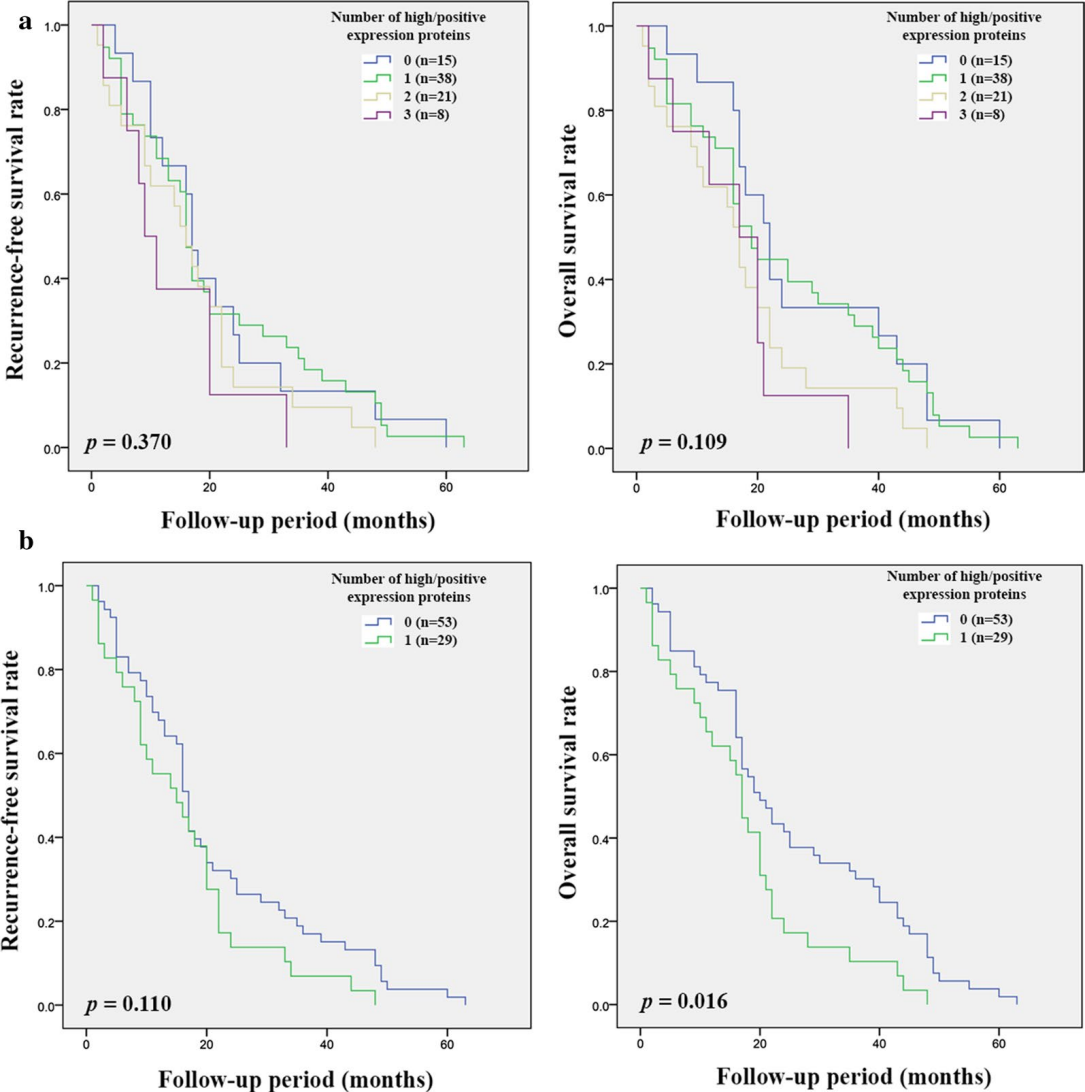
Kaplan–Meier analyses of recurrence-free and overall survival on the combination of CD44, CD44v6, and CD44v8-10 expression in extrahepatic CCA patients. **a** Recurrence-free and overall survival of patients according to the number of high or positive expression proteins. **b** Recurrence-free and overall survival of patients with zero or one high or positive expression marker versus high or positive expression of two or three markers

CD44, CD44v6, CD44v8-10 and ALDH1A1 showed prognostic significance for CCA patients. The correlation between these markers was therefore further analyzed and significant positive correlations between CD44, CD44v6, and CD44v8-10 were observed, while there was no significant correlation between ALDH1A1 with the other markers (Table 5). The combination of high expression of CD44 with positive expression of CD44v6 and CD44v8-10 was significantly associated with RFS (*p* = 0.001 and *p* = 0.002) and OS (*p* = 0.001 and *p* < 0.001) in intrahepatic CCA. Patients with high or positive expression of two or three markers had a poorer prognosis compared with other groups of patients (Fig. 5a and 5b). On the other hand, only high expression of CD44 with a positive expression of CD44v6 and CD44v8-10 was significantly associated with OS (*p* = 0.016) (Fig. 6b).

**Table 5:** The correlation coefficients between immunohistochemistry result of CD44, CD44v6, CD44v8-10, and ALDH1A1 in human CCA tissues

		CD44	CD44v6	CD44v8-10	ALDH1A1
CD44	Correlation coefficient	1	0.203	0.170	− 0.018
	*p*		*0.007*	*0.023*	0.816
CD44v6	Correlation coefficient		1	0.394	− 0.014
	*p*			*< 0.001*	0.849
CD44v8-10	Correlation coefficient			1	− 0.077
	*p*				0.849
ALDH1A1	Correlation coefficient				1
	*p*				

*CD44* cluster of differentiation 44, *CD44v* CD44 variant, *ALDH1A1* aldehyde dehydrogenase 1A1

### The correlation of soluble CSC markers with cancer recurrence

**Fig. 7:**
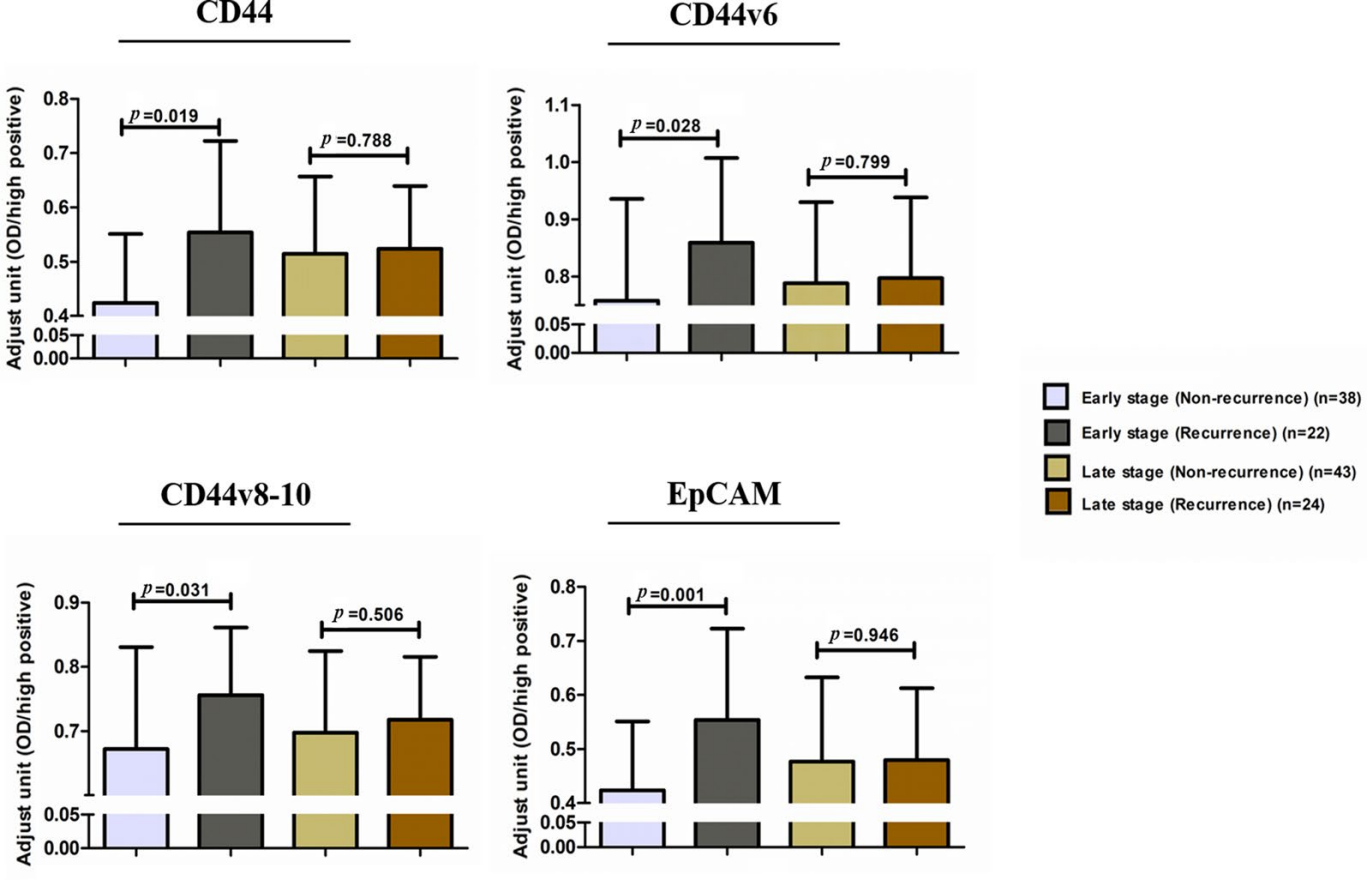
The different levels of soluble CD44, CD44v6, CD44v8-10, and EpCAM in patients with and without recurrence according to tumor staging. Adjusted units were calculated from optical density (OD) sample/OD high positive sample

The previous results showed that CD44, CD44v6, and CD44v8-10 were significantly correlated with RFS. In order to identify soluble CSC markers that can be used to predict cancer relapse, soluble CD44, CD44v6, and CD44v8-10 were further determined in CCA sera. Moreover, soluble EpCAM was also investigated because there is considerable evidence suggesting that this plays an important role in the progression of many cancers. In addition, the result of IHC showed that T stage, the present of lymph node metastasis and TNM staging were associated with RFS and OS. Therefore, the different of soluble CSC markers, CD44, CD44v6, CD44v8-10 and EpCAM on patients with and without recurrence was analyzed according to tumor staging in order to avoid the effect of T, N and TNM stage on recurrence. The detailed information of 127 sera CCA samples was summarized in Additional file 1: Table S1. The result showed that patients with early stage CCA had levels of soluble CSC markers, CD44, CD44v6, CD44v8-10, and EpCAM that were significantly increased in patients with recurrence (*p* = 0.019, *p* = 0.028, *p* = 0.031, and *p* = 0.001). On the other hand, there were no differences in soluble CSC markers between patients with recurrence and those without recurrence in the late stage (Fig. 7).

### Correlation between CSC marker levels in sera with clinicopathological features and laboratory results

The correlation between the levels of soluble CSC markers with clinicopathological features and laboratory results was analyzed. The results from the early stage group show that high levels of CD44, CD44v8-10 and EpCAM were significantly correlated with high levels of CA19-9 (*p* = 0.006, *p* = 0.011 and *p* < 0.001, respectively) (Table 6). On the other hand, there was no correlation found between sex, age, tumor site, histology type, CEA, AFP and liver function test. In addition, the results from the late stage group show that a high level of CD44v6 was significantly associated with elevated total bilirubin, direct bilirubin, AST and ALP (*p* = 0.037, *p* = 0.029, *p* = 0.037 and *p* = 0.049, respectively) (Table 7). Moreover, CD44v8-10 and EpCAM were also associated with elevated of ALP (*p* = 0.024 and *p* = 0.006) (Table 7).

**Table 6:** The correlation between CSC marker levels in sera with clinicopathological features and laboratory results in early stage CCA patients

Variables	Early stage (TNM stage I, II)								
	n	CD44	*p*	CD44v6	*p*	CD44v8-10	*p*	EpCAM	*p*
Sex									
Female	22	0.47 ± 0.13		0.80 ± 0.17		0.70 ± 0.13		0.46 ± 0.16	
Male	38	0.51 ± 0.16	0.306	0.79 ± 0.18	0.861	0.70 ± 0.15	0.913	0.48 ± 0.15	0.623
Age (year)									
Less than 61	30	0.51 ± 0.15		0.82 ± 0.15		0.72 ± 0.12		0.50 ± 0.15	
61 or greater	30	0.49 ± 0.16	0.579	0.77 ± 0.20	0.220	0.68 ± 0.17	0.276	0.44 ± 0.16	0.102
Tumor site									
Intrahepatic	31	0.50 ± 0.13		0.80 ± 0.15		0.71 ± 0.13		0.47 ± 0.13	
Extrahepatic	29	0.49 ± 0.18	0.888	0.79 ± 0.20	0.778	0.70 ± 0.17	0.710	0.47 ± 0.18	0.928
Histology type									
Papillary	38	0.52 ± 0.13		0.82 ± 0.14		0.72 ± 0.13		0.48 ± 0.16	
Others	22	0.47 ± 0.18	0.229	0.74 ± 0.21	0.079	0.68 ± 0.17	0.277	0.45 ± 0.16	0.449
Tumor marker									
CA19-9 (U/mL)									
< 37	24	0.44 ± 0.13		0.77 ± 0.16		0.67 ± 0.14		0.40 ± 0.11	
≥ 37	21	0.57 ± 0.15	*0.006*	0.85 ± 0.13	0.072	0.77 ± 0.10	*0.011*	0.54 ± 0.14	*< 0.001*
CEA (ng/mL)									
< 2.5	16	0.48 ± 0.12		0.81 ± 0.15		0.70 ± 0.14		0.44 ± 0.12	
≥ 2.5	30	0.52 ± 0.16	0.405	0.81 ± 0.15	0.937	0.72 ± 0.13	0.653	0.48 ± 0.15	0.286
AFP (IU/mL)									
< 5	33	0.48 ± 0.15		0.79 ± 0.14		0.70 ± 0.13		0.45 ± 0.14	
≥ 5	4	0.55 ± 0.21	0.387	0.80 ± 0.14	0.894	0.71 ± 0.13	0.937	0.50 ± 0.21	0.553
Liver function test									
Direct bilirubin (mg/dL)									
< 1.5	35	0.48 ± 0.14		0.79 ± 0.15		0.69 ± 0.13		0.44 ± 0.13	
≥ 1.5	18	0.53 ± 0.19	0.261	0.79 ± 0.22	0.937	0.71 ± 0.18	0.600	0.49 ± 0.17	0.276
Total bilirubin (mg/dL)									
< 2.5	33	0.49 ± 0.14		0.80 ± 0.15		0.70 ± 0.14		0.46 ± 0.13	
≥ 2.5	22	0.51 ± 0.18	0.571	0.78 ± 0.21	0.696	0.70 ± 0.17	0.866	0.46 ± 0.17	0.992
ALT (U/L)									
< 40	30	0.50 ± 0.14		0.81 ± 0.16		0.73 ± 0.12		0.46 ± 0.11	
≥ 40	23	0.48 ± 0.18	0.521	0.75 ± 0.19	0.238	0.66 ± 0.17	0.089	0.46 ± 0.19	0.888
AST (U/L)									
< 40	28	0.48 ± 0.13		0.79 ± 0.16		0.70 ± 0.13		0.47 ± 0.13	
≥ 40	25	0.50 ± 0.18	0.709	0.78 ± 0.19	0.794	0.69 ± 0.17	0.752	0.46 ± 0.17	0.834
ALP (U/L)									
< 130	18	0.45 ± 0.15		0.76 ± 0.16		0.68 ± 0.13		0.42 ± 0.12	
≥ 130	35	0.52 ± 0.16	0.129	0.80 ± 0.18	0.479	0.71 ± 0.16	0.575	0.48 ± 0.16	0.151

*CD44* cluster of differentiation 44, *CD44v* CD44 variant, *EpCAM* epithelial cell adhesion molecules, *TNM* primary tumor-node-metastasis, *CA19-9* cancer antigen 19-9, *CEA* carcinoembryonic antigen, *AFP* alpha-fetoprotein, *ALT* alanine aminotransferase, *AST* aspartate aminotransferase, *ALP* alkaline phosphatase

**Table 7:** The correlation between CSC marker levels in sera with clinicopathological features and laboratory results in late stage CCA patients

Variables	Late stage (TNM stage III, IV)								
	n	CD44	*p*	CD44v6	*p*	CD44v8-10	*p*	EpCAM	*p*
Sex									
Female	24	0.51 ± 0.16		0.80 ± 0.15		0.71 ± 0.12		0.50 ± 0.17	
Male	43	0.52 ± 0.12	0.584	0.78 ± 0.14	0.573	0.70 ± 0.12	0.940	0.47 ± 0.13	0.435
Age (year)									
Less than 61	32	0.51 ± 0.12		0.80 ± 0.12		0.69 ± 0.10		0.46 ± 0.14	
61 or greater	35	0.52 ± 0.14	0.769	0.78 ± 0.16	0.651	0.71 ± 0.13	0.505	0.50 ± 0.16	0.289
Tumor site									
Intrahepatic	38	0.50 ± 0.13		0.77 ± 0.15		0.70 ± 0.11		0.46 ± 0.12	
Extrahepatic	29	0.54 ± 0.13	0.231	0.82 ± 0.13	0.128	0.71 ± 0.13	0.539	0.50 ± 0.17	0.259
Histology type									
Papillary	29	0.51 ± 0.14		0.78 ± 0.15		0.70 ± 0.12		0.47 ± 0.17	
Others	38	0.53 ± 0.13	0.599	0.80 ± 0.14	0.510	0.71 ± 0.12	0.809	0.49 ± 0.13	0.562
Tumor marker									
CA19-9 (U/mL)									
< 37	13	0.47 ± 0.14		0.79 ± 0.13		0.71 ± 0.13		0.44 ± 0.13	
≥ 37	33	0.53 ± 0.13	0.152	0.80 ± 0.16	0.828	0.70 ± 0.11	0.841	0.48 ± 0.13	0.257
CEA (ng/mL)									
< 2.5	6	0.51 ± 0.20		0.71 ± 0.19		0.71 ± 0.10		0.52 ± 0.09	
≥ 2.5	37	0.51 ± 0.11	0.888	0.80 ± 0.13	0.171	0.69 ± 0.10	0.598	0.44 ± 0.11	0.108
AFP (IU/mL)									
< 5	33	0.52 ± 0.14		0.79 ± 0.16		0.70 ± 0.11		0.46 ± 0.11	
≥ 5	4	0.45 ± 0.20	0.357	0.77 ± 0.11	0.792	0.61 ± 0.06	0.118	0.38 ± 0.11	0.199
Liver function test									
Direct bilirubin (mg/dL)									
< 1.5	39	0.50 ± 0.14		0.77 ± 0.16		0.70 ± 0.11		0.46 ± 0.13	
≥ 1.5	17	0.56 ± 0.12	0.137	0.85 ± 0.09	*0.029*	0.71 ± 0.11	0.632	0.51 ± 0.14	0.227
Total bilirubin (mg/dL)									
< 2.5	35	0.49 ± 0.14		0.76 ± 0.16		0.68 ± 0.11		0.46 ± 0.13	
≥ 2.5	21	0.55 ± 0.12	0.089	0.85 ± 0.11	*0.037*	0.73 ± 0.11	0.149	0.51 ± 0.13	0.181
ALT (U/L)									
< 40	28	0.49 ± 0.14		0.76 ± 0.16		0.70 ± 0.12		0.48 ± 0.14	
≥ 40	28	0.54 ± 0.12	0.148	0.83 ± 0.13	0.091	0.70 ± 0.11	0.946	0.47 ± 0.12	0.708
AST (U/L)									
< 40	25	0.49 ± 0.15		0.75 ± 0.18		0.68 ± 0.11		0.45 ± 0.12	
≥ 40	31	0.54 ± 0.12	0.150	0.83 ± 0.11	*0.037*	0.72 ± 0.11	0.187	0.49 ± 0.14	0.295
ALP (U/L)									
< 130	19	0.47 ± 0.13		0.74 ± 0.16		0.65 ± 0.11		0.41 ± 0.11	
≥ 130	37	0.54 ± 0.13	0.056	0.82 ± 0.13	*0.049*	0.72 ± 0.11	*0.024*	0.51 ± 0.13	*0.006*

*CD44* cluster of differentiation 44, *CD44v* CD44 variant, *EpCAM* epithelial cell adhesion molecules, *TNM* primary tumor-node-metastasis, *CA19*-9 cancer antigen 19-9, *CEA* carcinoembryonic antigen, *AFP* alpha-fetoprotein, *ALT* alanine aminotransferase, *AST* aspartate aminotransferase, *ALP*: alkaline phosphatase

### Predictive value of soluble CSC markers for post-operative recurrence

The receiver operator characteristic (ROC) curve was analyzed according tumor staging. The cut-off values for soluble CD44, CD44v6, CD44v8-10 and EpCAM suitable for the discrimination between recurrence and non-recurrence in the patients with early stage CCA were 0.505 (area under curve; AUC = 0.670, *p* = 0.029), 0.814 (AUC = 0.670, *p* = 0.029), 0.713 (AUC = 0.702, *p* = 0.010), and 0.506 (AUC = 0.739, *p* = 0.002), respectively (Additional file 2: Fig. S1). The sensitivity and specificity to distinguish between recurrence and non-recurrence are shown in Table 8. The positive predictive value (PPV) and negative predictive value (NPV) were 51.7 and 77.4 for CD44, 55.6 and 78.8 for CD44v6, 59.3 and 81.8 for CD44v8-10 and 62.5 and 80.6 for EpCAM. By using the cut-off derived from the ROC curve, the predictive ability of CD44, CD44v6, CD44v8-10 and EpCAM on post-operative recurrence was explored. The crude and adjusted odds ratio (OR) of CD44, CD44v6, CD44v8-10 and EpCAM were 3.67 (*p* = 0.022), 4.64 (*p* = 008), 6.55 (*p* = 0.002), 6.91 (*p* = 0.001) and 3.62 (*p* = 0.031), 4.98 (*p* = 0.009), 5.92 (*p* = 0.004), 6.23 (*p* = 0.003) (Table 9). On the other hand, soluble CD44, CD44v6, CD44v8-10, and EpCAM were not suitable to distinguish between recurrence and non-recurrence in patients with late stage CCA (Additional file 3: Fig. S2).

**Table 8:** Predictive values for soluble CD44, CD44v6, CD44v8-10 and EpCAM for predicting CCA recurrence using the optimal cut-off derived from the ROC curve in early stage of CCA patients

Variables	AUC (95% CI)	Cut-off	Sensitivity (%)	Specificity (%)	PPV (%)	NPV (%)	*p*
CD44	0.670 (0.521–0.819)	0.505	68.0	63.0	51.7	77.4	*0.029*
CD44v6	0.670 (0.529–0.811)	0.814	68.0	68.0	55.6	78.8	*0.029*
CD44v8-10	0.702 (0.563–0.841)	0.713	73.0	71.0	59.3	81.8	*0.010*
EpCAM	0.739 (0.598–0.880)	0.506	68.0	76.0	62.5	80.6	*0.002*

*CD44* cluster of differentiation 44, *CD44v* CD44 variant, *EpCAM* epithelial cell adhesion molecules, *AUC* area under curve, *95% CI* 95% confidence interval, *PPV* positive predictive value, *NPV* negative predictive value

**Table 9:** Predictive risk of CCA recurrence in early stage patients using soluble CD44, CD44v6, CD44v8-10, and EpCAM

Comparative prediction	Post-operative recurrence			
	OR crude	*p* (95% CI)	OR adjusted	*p* (95% CI)
CD44 ≥ 0.505 vs. < 0.505	3.67	*0.022* (1.207–11.183)	3.62	*0.031* (1.126–11.66)
CD44v6 ≥ 0.814 vs. < 0.814	4.64	*0.008* (1.503–14.346)	4.98	*0.009* (1.504–16.473)
CD44v8-10 ≥ 0.713 vs. < 0.713	6.55	*0.002* (2.029-21.116)	5.92	*0.004* (1.779-19.706)
EpCAM ≥ 0.814 vs. < 0.814	6.91	*0.001* (2.147–22.202)	6.23	*0.003* (1.867–20.793)

*CD44* cluster of differentiation 44, *CD44v* CD44 variant, *EpCAM* epithelial cell adhesion molecules, *OR* odds ratio, *OR adjusted* odds ratio adjusted for age and sex statistical analysis, *95% CI* 95% confidence interval

### The combination of soluble CSC markers and CA19-9 for improving predictive ability for post-operative recurrence

**Fig. 8:**
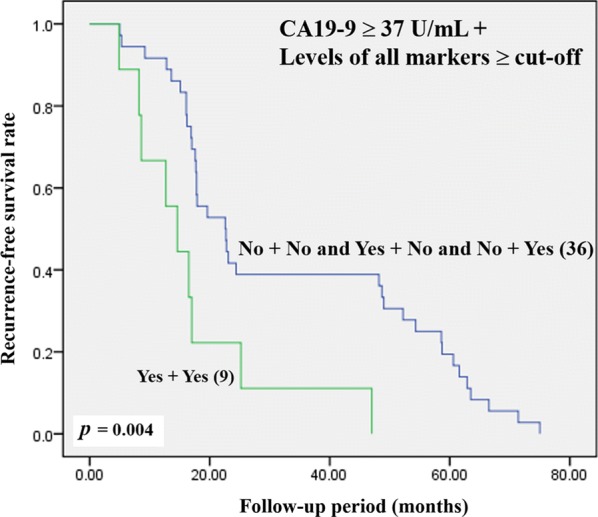
Kaplan–Meier analysis of recurrence-free survival on patients with high levels of CD44, CD44v6, CD44v8-10 and EpCAM with CA19-9 versus other groups of patients

Soluble CD44, CD44v6, CD44v8-10 and EpCAM are promising factors for predicting cancer recurrence. A combination of these markers and their predictive efficacy for cancer recurrence was further examined. Interestingly, a combination of high levels of CD44, CD44v6, CD44v8-10 and EpCAM could increase the risk for recurrence with a high value of crude OR (crude OR = 7.08, *p* = 0.004) and adjusted OR (adjusted OR = 7.39, *p* = 0.006). Moreover, the best predictive ability for recurrence was observed with the combination of high expression of these 4 CSC markers and elevated CA19-9 levels with an increase of the crude and adjusted OR to 12.25 (*p* = 0.005) and 15.28 (*p* = 0.011), respectively (Table 10). The survival analysis was also evaluated in patients with high levels of CD44, CD44v6, CD44v8-10 and EpCAM combined with an elevated CA19-9 level compared with other groups of patients. Patients with high levels of CD44, CD44v6, CD44v8-10 and EpCAM combined with elevated CA19-9 had a lower RFS when compared with other groups (*p *= 0.004) (Fig. 8).

**Table 10:** Predictive risk of CCA recurrence in early stage patients using either the combination of soluble CSC markers, CD44, CD44v6, CD44v8-10 and EpCAM or the combination of soluble CSC markers with CA19-9

Comparative prediction	OR	*p*	OR	*p*
	Crude	(95% CI)	Adjusted	(95% CI)
Post-operative recurrence				
Levels of all markers ≥ cut-off^a^				
No^b^ (46)	1	*0.004*	1	*0.006*
Yes (14)	7.08	(1.867–26.870)	7.39	(1.760–31.071)
CA19-9 ≥ 37 U/mL + Levels of all markers ≥ cut-off				
No + No and Yes + No and No + Yes (36)	1	*0.005*	1	*0.011*
Yes + Yes (9)	12.25	(2.114–70.986)	15.28	(1.879–124.320)

*OR* odds ratio, *OR adjusted* odds ratio adjusted for age and sex statistical analysis, *95% CI* 95% confidence interval

^a^Levels of all markers: levels of CD44, CD44v6, CD44v8-10 and EpCAM

^b^No: patients with at least one of marker lower than a designated cut-off

## Discussion

CCA is a malignant tumor with an asymptomatic early stage so that the disease is usually diagnosed once it has become advanced, resulting in a poor outcome for patients after treatment [23]. Even though several therapeutic approaches can be considered for CCA treatment, the recurrence rate is still high and leads to a high mortality in CCA patients [5]. Many studies suggest that tumor size and metastatic status are potential factors influencing RFS and OS in CCA patients [24–27]. Similar to our study, we found that CCA patients with a high primary tumor stage, presence of regional lymph node metastasis and high TNM staging have a lower RFS and OS compared with other groups of patients. Even though several studies have reported potential pathological factors for predicting CCA recurrence, effective biomarkers are required to assess the potential outcome of patients, including survival rate and the probability of recurrence after treatment. Moreover, the presence of such markers is likely to be useful for targeted therapy in order to prevent cancer progression and recurrence.

The subpopulation of cancer cells with stem cell-like properties, CSCs, has been reported to be involved in many cancer processes such as tumor growth, metastasis, resistance to treatment, as well as recurrence [28]. Raggi et al. demonstrated the existent of CSC in biliary tract cancer (BTC) and suggest that the isolated BTC cells that express CD24, CD44 or EpCAM had a higher potential of tumorigenesis than the negative groups [29]. In addition, other CSC markers have also been reported as markers for CSC in CCA [7]. Therefore, CSC markers might be used to predict CCA progression and recurrence. To answer this hypothesis, we performed immunohistochemical staining to evaluate the expression of 6 putative CSC markers, CD44, CD44v6, CD44v8-10, CD133, EpCAM and ALDH1A1 in CCA tissue. The results show that among the 6 CSC markers investigated, the expression of CD44 and its variant isoforms (CD44v6 and CD44v8-10), and also ALDH1A1, were associated with tumor progression and poor outcome of CCA patients, including short RFS and OS. CD44 is a well-known marker that plays an important role in tumor progression, but the different isoforms work differently [30]. There is considerable evidence suggesting that a high expression of CD44 is associated with tumor progression and recurrence [31, 32], which is similar to the other two variant isoforms that have also been reported to be involved in cancer progression and recurrence [33–35]. This is consistent with our finding for CCA which shows that patients with a high expression of CD44, CD44v6, and CD44v8-10 had a shorter RFS and OS compared with the low expression group. In addition, the expression of these markers seems to increase along with tumor stage, suggesting that their expression is involved in tumor progression. ALDH1A1 is cytosolic enzyme that can convert retinol into retinoic acid. It plays an important role in many processes occurring in the normal cell, include growth, development and differentiation [21]. It has been reported to be marker for normal stem cells (SC) and also for CSC. Although many studies have reported that a high expression of ALDH1A1 is associated with tumor progression, this result is controversial as many studies have shown that a high expression of ALDH1A1 is correlated with a favorable prognosis in different cancers [21]. In the present study, we found that a high expression of ALDH1A1 was also associated with a favorable prognosis for CCA patients. There is evidence suggesting that a combination of protein expressions has more potential to divide patients into the different prognostic groups [36]. Thus, the correlation of our 4 promising markers was also analyzed. A significant positive correlation was found in CD44, CD44v6 and CD44v8-10, with the combination of high expression in two or three markers being more useful in dividing patients into the different prognostic groups. On the other hand, there was no correlation between ALDH1A1 and the other markers.

The panel of protein expression markers (CD44, CD44v6, and CD44v8-10) shows more efficacy in discriminating patients into different prognostic groups than the individual markers. Moreover, the elevation of these markers was also associated with RFS. Therefore, we further investigated the levels of these markers in the serum using the ELISA technique so that it can be used diagnostically for predicting factors for CCA recurrence. As many studies suggest that soluble EpCAM is associated with an aggressive tumor phenotype [18–20], soluble EpCAM was also considered to be a marker for CCA recurrence. According to the literature, tumor staging is an important factor involved in tumor recurrence in CCA patients [27], and our results on IHC also demonstrate that tumor staging has the potential to predict CCA recurrence. Therefore, in order to determine the effect of staging on cancer recurrence, the different levels of soluble CD44, CD44v6, CD44v8-10 and EpCAM in patients with and without recurrence were examined according to staging. The results indicate that early stage tumors are less variable than late or advanced stage tumors. Thus, the recurrence of cancer is caused by the inherent resistance of cancer cells [37]. Our results on early stage CCA patients show that patients with a low T stage, absence of lymph node involvement and no distant metastases but with recurrence had higher soluble levels of CD44, CD44v6, CD44v8-10 and EpCAM compared with those patients without recurrence. Accumulating evidence indicates that highly proliferative cancer cells can be killed by chemotherapy and radiotherapy, however a subpopulation of cancer cells with therapeutic resistance might survive and lead to relapse [6]. CD44 is known as a surface marker associated with CSC in various cancer types, and several CD44 variant isoforms are generated by alternative splicing processes [38]. Shi et al. reported that the expression of CD44v6 is up-regulated in the recurrence ovarian cancer, and this is also associated with cancer progression and metastasis [34]. Another CSC marker, CD44v8-10 stabilizes xCT, which is a cystine–glutamate transporter inducing glutathione synthesis. This process contributes to the tumor cells becoming resistant to oxidative stress, including reactive oxygen species (ROS) [39]. In addition, a study by Tayama et al. on ovarian cancer demonstrated that chemotherapy mostly eliminated the EpCAM-negative population compared with the EpCAM-positive population, suggesting that the EpCAM-positive population contributes to chemoresistance and cancer recurrence after chemotherapy [40]. Thus, the CSC markers, CD44, CD44v6, CD44v8-10, and EpCAM have the potential to predict cancer recurrence including CCA.

The levels of tumor markers (CA19-9, CEA, and AFP) and a liver function test were also used to monitor CCA patients after treatment [27]. In this study, we also found that high levels of soluble CD44, CD44v6, CD44v8-10 and EpCAM were correlated with elevated levels of CA19-9, suggesting that their expression is involved in tumor progression. However, in late stage disease, there was no difference in the levels of soluble CD44, CD44v6, CD44v8-10 and EpCAM in patients with and without recurrence, even though some of them showed an association with poor results for the liver function test. Therefore, our further analysis focused on early stage disease in CCA patient with the aim of examining the predictive value of soluble CD44, CD44v6, CD44v8-10 and EpCAM on post-operative CCA recurrence. Interestingly, we found that either high levels of soluble CD44, CD44v6, CD44v8-10 and EpCAM alone or a combination of these markers provides more precise predictive potential of CCA recurrence. Furthermore, there are studies that suggest that elevated serum levels of CA19-9 are also associated with CCA recurrence [27, 41], a result corroborated by our study with soluble CD44, CD44v6, CD44v8-10, EpCAM and CA19-9. Therefore, the association between the combination of high levels of these 4 markers and CA19-9 was further evaluated. Our findings suggest that overexpression of the panel of CSC markers in combination with elevated levels of CA19-9 provide the best predictive factor for the post-operative recurrence of CCA in early stage patients. However, the small number of patients is a limitation of this study and a larger independent patient cohort needs to be further evaluated before clinical application.

## Conclusion

The elevated of CD44, CD44v6, CD44v8-10 and EpCAM increases predictability of post-operative CCA recurrence. Moreover, the best predictive ability was found in the combination of overexpression of the panel of CSC markers with CA19-9.

## Supplementary information

**Additional file 1: Table S1.** The detailed information of sera CCA samples.

**Additional file 2: Fig S1.** ROC curve of soluble CD44, CD44v6, CD44v8-10, and EpCAM for predicting CCA recurrence in early stage CCA patients. AUC represents the area under curve for each protein.

**Additional file 3: Fig S2.** ROC curve of soluble CD44, CD44v6, CD44v8-10, and EpCAM for predicting CCA recurrence in late stage CCA patients. AUC represents the area under curve for each protein.

## Data Availability

The datasets generated during and/or analyzed during the current study are available from the corresponding author on reasonable request.

## Abbreviations

ALDH1A1: Aldehyde dehydrogenase 1A1
CCA: Cholangiocarcinoma
CSC: Cancer stem cell
CD44: Cluster of differentiation 44
CD44v6: Cluster of differentiation 44 variant 6
CD44v8-10: Cluster of differentiation 44 variants 8-10
CD133: Cluster of differentiation 133
EpCAM: Epithelial cell adhesion molecule
CA19-9: Carbohydrate antigen
CEA: Carcinoembryonic antigen
OS: Overall survival
RFS: Recurrence-free survival
