# Supplementary material for: Overexpression of a panel of cancer stem cell markers enhances the predictive capability of the progression and recurrence in the early stage cholangiocarcinoma
Source: J Transl Med. 2020 Feb 10;18:64. doi: 10.1186/s12967-020-02243-w (PMC7008521; doi:10.1186/s12967-020-02243-w)
Supplement: Supplementary file 1 — Additional file 1: Table S1. The detailed information of sera CCA samples. [file 12967_2020_2243_MOESM1_ESM.docx]

**Table S1.** The detailed information of sera CCA samples

| **Variables** | **Number** | **%** |
| --- | --- | --- |
| Number of patients | 127 | 100 |
| Sex  Female  Male | 46  81 | 36  64 |
| Age (year)  Less than 61  61 or greater | 62  65 | 49  51 |
| Tumor site  Intrahepatic  Extrahepatic | 69  58 | 54  46 |
| Histology type  Papillary  Others | 67  60 | 53  47 |
| Primary tumor (T)  I, II  III, IV | 83  44 | 65  35 |
| Lymph nodes (N) metastasis  No  Yes | 79  48 | 62  38 |
| Distant metastasis (M)  No  Yes | 124  3 | 98  2 |
| TNM Stage  I, II  III, IV | 60  67 | 47  53 |
| TNM: primary tumor-node-metastasis | | |
