# Supplementary figures and images for: Overexpression of a panel of cancer stem cell markers enhances the predictive capability of the progression and recurrence in the early stage cholangiocarcinoma
Source: J Transl Med. 2020 Feb 10;18:64. doi: 10.1186/s12967-020-02243-w (PMC7008521; doi:10.1186/s12967-020-02243-w)

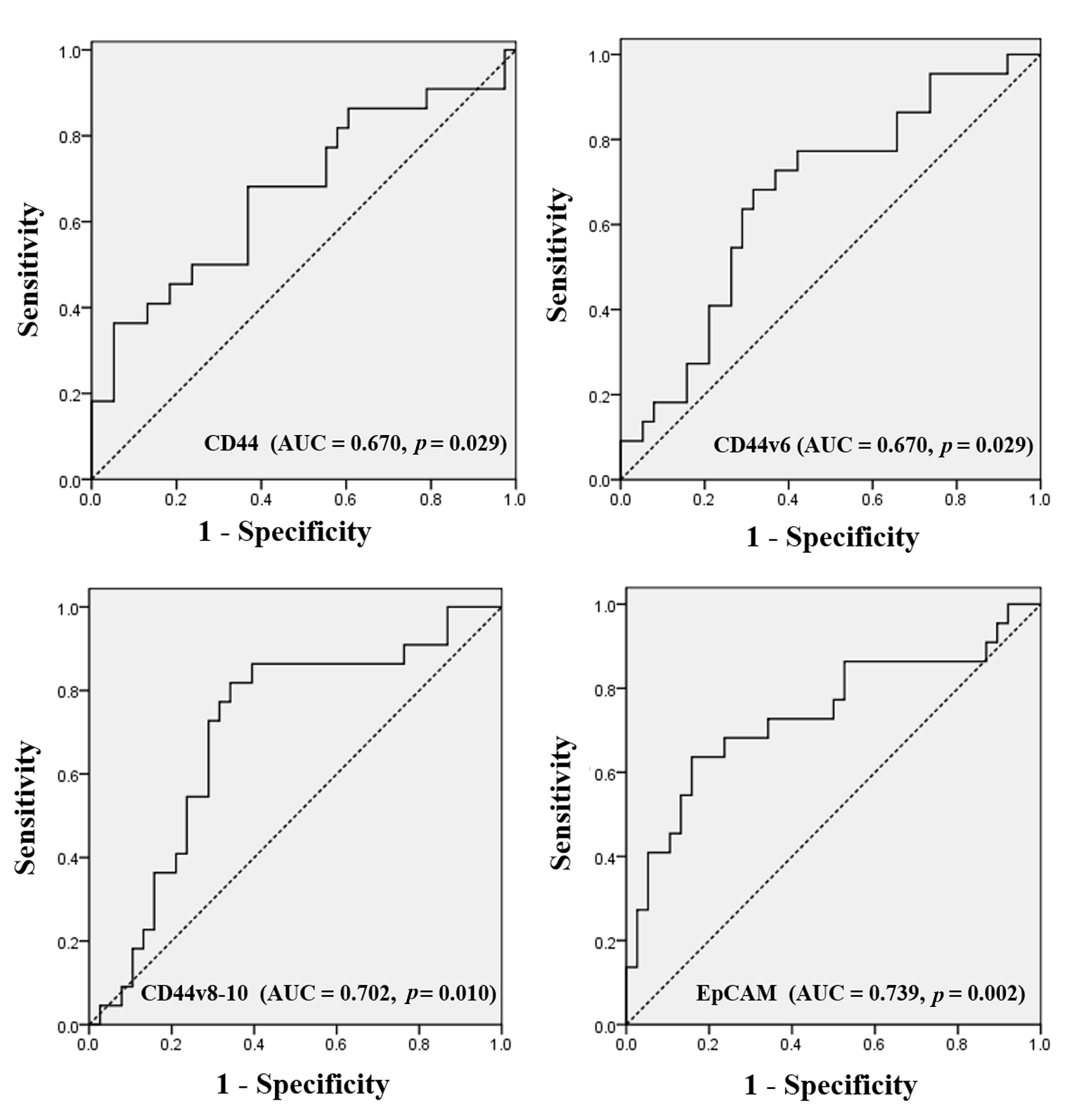

Supplement: Supplementary file 2 — Additional file 2: Fig S1. ROC curve of soluble CD44, CD44v6, CD44v8-10, and EpCAM for predicting CCA recurrence in early stage CCA patients. AUC represents the area under curve for each protein. [file 12967_2020_2243_MOESM2_ESM.tif]

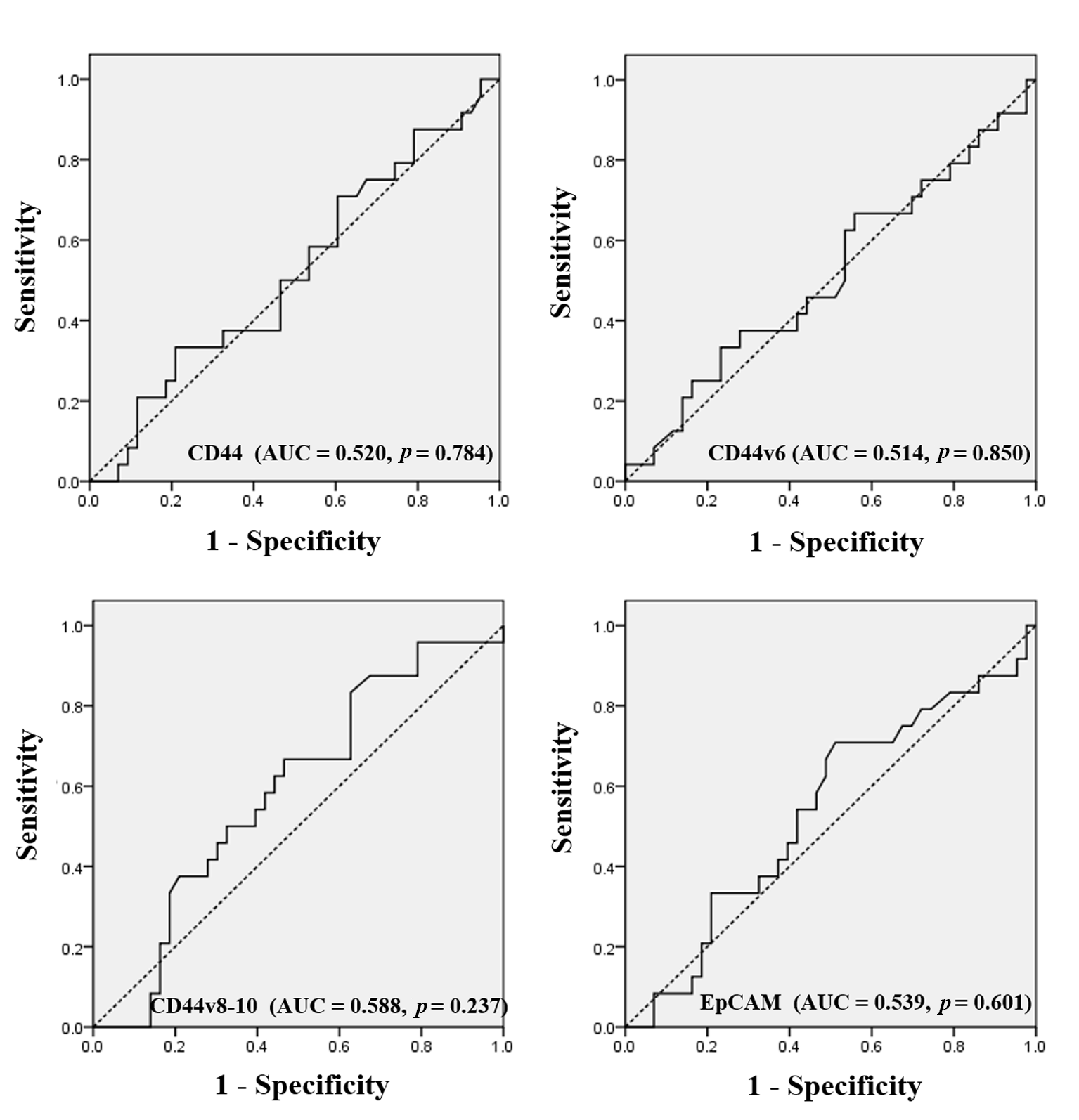

Supplement: Supplementary file 3 — Additional file 3: Fig S2. ROC curve of soluble CD44, CD44v6, CD44v8-10, and EpCAM for predicting CCA recurrence in late stage CCA patients. AUC represents the area under curve for each protein. [file 12967_2020_2243_MOESM3_ESM.tif]
